# Supplementary material for: Complexome profiling of the Chlamydomonas psb28 mutant reveals TEF5 as an early PSII assembly factor
Source: Plant Cell. 2025 Mar 17;37(6):koaf055. doi: 10.1093/plcell/koaf055 (PMC12164590; doi:10.1093/plcell/koaf055)
Supplement: koaf055_Supplementary_Data [file koaf055_supplementary_data.zip › psb28_interactive.zip!/plots/intensity/qualityPlots/peptides/_Cre06.g294450.t1.1.html]

### Cre06.g294450.t1.1

Trivial name:   
SeqCoverage [median]: 17.40  
Mass: 40.20 kDa  
  
 

| Sequence | MissedCleavage | Mass [Da] | wtIntensity | muIntensity | Score |
| --- | --- | --- | --- | --- | --- |
| EQAPDLPRPVVATK | 1 | 1519.84 | 0.00 | 2835.69 | 42.09 |
| FTAASVTAAAK | 0 | 1036.56 | 2942.97 | 2980.42 | 81.52 |
| LSEGEIAELDRVSAK | 1 | 1615.84 | 625.14 | 0.00 | 39.01 |
| LTEVAPVIEAVR | 0 | 1295.74 | 0.00 | 534.15 | 101.11 |
| RLTEVAPVIEAVR | 1 | 1451.85 | 1822.87 | 0.00 | 52.26 |
| YSASGAKPSGPR | 1 | 1176.59 | 613.13 | 0.00 | 41.40 |
|  |  |  | 6004.1 | 6350.3 |  |
